# Ovarian Cancer: Current Treatment and Patient Management

**Published:** 2016-04-01

**Authors:** Bradley J. Monk, Paula J. Anastasia

**Affiliations:** University of Arizona Cancer Center, Phoenix, Arizona, and Cedars-Sinai Medical Center, Los Angeles, California

The decisions made by patients with ovarian cancer are tough ones. They affect the entire treatment paradigm, have long-lasting ramifications, and should be made through a collaboration between patients and clinicians, according to Bradley J. Monk, MD, of the University of Arizona Cancer Center, Phoenix, Arizona, and Paula J. Anastasia, RN, MS, AOCN®, of Cedars-Sinai Medical Center in Los Angeles, California, who spoke on the topic at JADPRO Live.

Dr. Monk and Ms. Anastasia discussed the rationale behind front-line treatment decisions in advanced ovarian cancer, addressed quality-of-life concerns, and emphasized the importance of genetic testing.

Patients should be carefully guided through a discussion of these different treatment strategies, according to Ms. Anastasia. Such complex conversations are best accomplished one step at a time, she maintained. Discussions about advanced disease should center upon survival in the context of hope and realistic goals. "There is a high probability of achieving a remission—not a cure—with chemotherapy," she emphasized.

Women with advanced ovarian cancer have a range of options, and no one treatment is right for every patient. For example, each woman’s cancer comprises certain cell types and molecular abnormalities, such as KRAS mutations in mucinous tumors and BRCA1/2 deficiencies in high-grade serous tumors. Treatments must be designed with these and other individual characteristics in mind.

Options include surgical resection, neoadjuvant chemotherapy, weekly chemotherapy, intraperitoneal chemotherapy, and in some cases, targeted agents. The choice among them reflects the art of personalized medicine, the speakers agreed.

## SURGERY IS KEY

Dr. Monk emphasized the importance of surgery in newly diagnosed advanced cancer. "The goal of primary surgery is complete resection; if you don’t resect all the cancer, it really doesn’t do any benefit," he pointed out.

Neoadjuvant chemotherapy and interval debulking after three cycles of chemotherapy may be the best initial approach in about half of patients, based on data from the CHORUS trial (Kehoe et al., 2013; Kehoe et al., 2015). According to Dr. Monk, neoadjuvant chemotherapy in patients with high tumor volume is at least as effective as primary surgery followed by chemotherapy, and perhaps better.

## CHEMOTHERAPY

Chemotherapy delivered weekly (vs. a conventional schedule) has gained some attention, based on data from the JGOG 3016 trial of dose-dense weekly paclitaxel with carboplatin (Katsumata et al., 2013). The hematologic toxicity of this approach, however, is worrisome, according to Ms. Anastasia, who indicated that dose-dense chemotherapy can be inconvenient for the patient and produce dose-limiting anemia and fatigue; this can require blood transfusions and can negatively impact quality of life, including sexuality.

**Figure 1:**
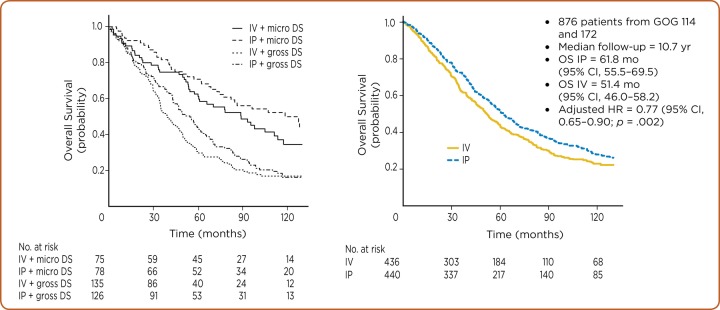
Long-term survival advantage and prognostic factors associated with IP chemotherapy treatment in advanced ovarian cancer: A GOG study. Information from Tewari et al. (2015).)

Intraperitoneal chemotherapy has shown benefits over intravenous chemotherapy, and according to a recent update of a key clinical trial, these advantages can extend beyond 10 years (Tewari et al., 2015; Figure 1). Dr. Monk indicated that intraperitoneal chemotherapy is best for patients with little residual tumor; it is not a good choice after an inadequate surgical resection. Ms. Anastasia emphasized that this is a tough form of treatment for many patients—it is toxic, time-consuming, and can compromise quality of life. Fewer than half of patients who begin this treatment are able to complete it, she noted, adding, "It is right for some people, but they have to be optimally debulked."

## TARGETED THERAPIES

Angiogenesis has proved to be a promising target in advanced ovarian cancer. The addition of the antiangiogenesis agent bevacizumab (Avastin) to front-line treatment remains controversial in the United States, but it has been approved in Europe based on data from the GOG 218 and ICON-7 trials (Burger et al., 2011; Perren et al., 2011). GOG 218 evaluated bevacizumab in combination with carboplatin and paclitaxel and showed a survival advantage in patients with a high tumor load, though the outcome proved insufficient to lead to regulatory approval of the drug. Dr. Monk and Ms. Anastasia agreed that due to multiple recurrences of their disease, most patients will need to receive bevacizumab at some point, if not off-label in the front-line setting.

## TREATMENT OF RECURRENT DISEASE

In the relapse setting, treatment considerations include the disease-free interval, existing toxicities from first-line treatment, volume of disease at the time of relapse, and serologic relapse (CA-125 measurement). Bevacizumab has a clear role here, they said.

For patients with platinum-refractory or platinum-resistant disease and recurrence within 6 months from last treatment, a nonplatinum agent, with or without bevacizumab, is indicated. For those with platinum-sensitive disease and recurrence beyond 6 months, a chemotherapy doublet with or without bevacizu-mab is indicated.

"You have to keep a score card on what the platinum-free interval is," Dr. Monk said. "If the patient recurs in less than 6 months, she can get bevacizumab on-label in the second and third line."

**Figure 2:**
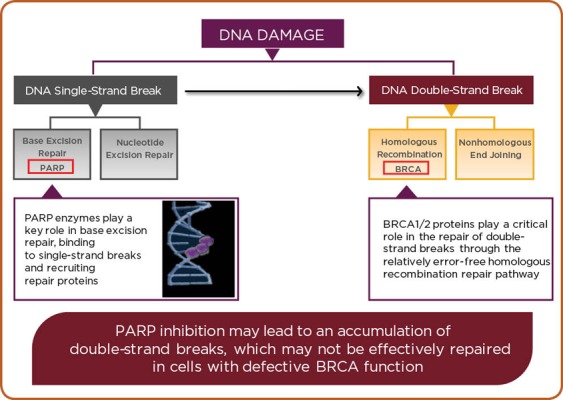
PARP and BRCA in DNA repair. Information from Hoeijmakers (2001); Polyak and Garber (2011); Underhill, Toulmonde, and Bonnefoi et al. (2011).

Single-agent treatment with the PARP inhibitor olaparib (Lynparza) is FDA-approved for patients with deleterious or suspected deleterious germline BRCA-mutated advanced ovarian cancer after three or more lines of chemotherapy (Figure 2). The availability of this targeted agent mandates that all patients be tested for BRCA status, according to Dr. Monk.

The National Comprehensive Cancer Network, the American Society of Clinical Oncology, and the Society of Gynecologic Oncologists recommend genetic testing for all women with ovarian, fallopian tube, and peritoneal carcinoma.

## CONCLUDING THOUGHTS

Finally, the speakers agreed that symptom palliation is important from the start of the treatment journey. Ongoing discussion between patients and their providers "will help get patients through today," Ms. Anastasia said. They emphasized as well that ovarian cancer is a chronic disease, and patients receive many lines of therapy. Treatment options beyond three lines remains an unmet clinical need.
